# Supplementary material for: Constitutive Activation of Guanylate Cyclase by the G86R GCAP1 Variant Is Due to “Locking” Cation-π Interactions that Impair the Activator-to-Inhibitor Structural Transition
Source: Int J Mol Sci. 2020 Jan 23;21(3):752. doi: 10.3390/ijms21030752 (PMC7037459; doi:10.3390/ijms21030752)
Supplement: Supplementary file 1 [file ijms-21-00752-s001.pdf]

International Journal of
Molecular Sciences

MDPI

Supplementary Materials

Constitutive Activation of Guanylate Cyclase by the G86R GCAP1 Variant is Due to "Locking" Cation-π Interactions that Impair the Activator-to-Inhibitor Structural Transition

Seher Abbas [1,#], Valerio Marino [2,#], Laura Bielefeld [3], Karl-Wilhelm Koch [4], and Daniele Dell'Orco [5,*]

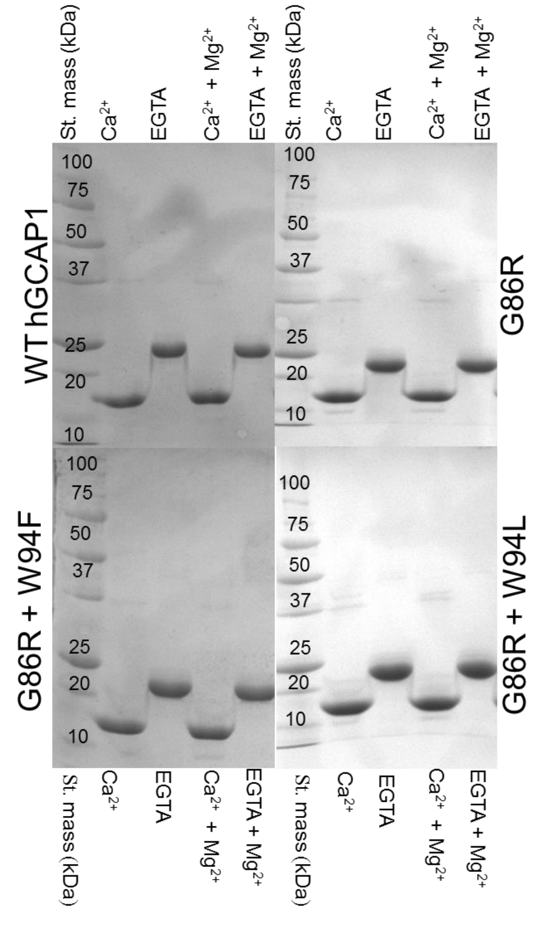

Figure S1. Ca^{2+} and Mg^{2+} -induced electrophoretic mobility shift of GCAP1 variants. Each GCAP1 variant (WT, G86R, G86R+W94F, G86R+W94L) was dissolved in 50 mM Tris/HCl pH 8.0 and incubated for 10 min at RT with either 1 mM Ca^{2+}, 1 mM EGTA, 1 mM Ca^{2+} and 1 mM Mg^{2+} or 1 mM EGTA and 1 mM Mg^{2+}. For each sample, 5 µg protein was loaded on a 15% SDS-PAGE gel. Mass standards are shown in kDa.

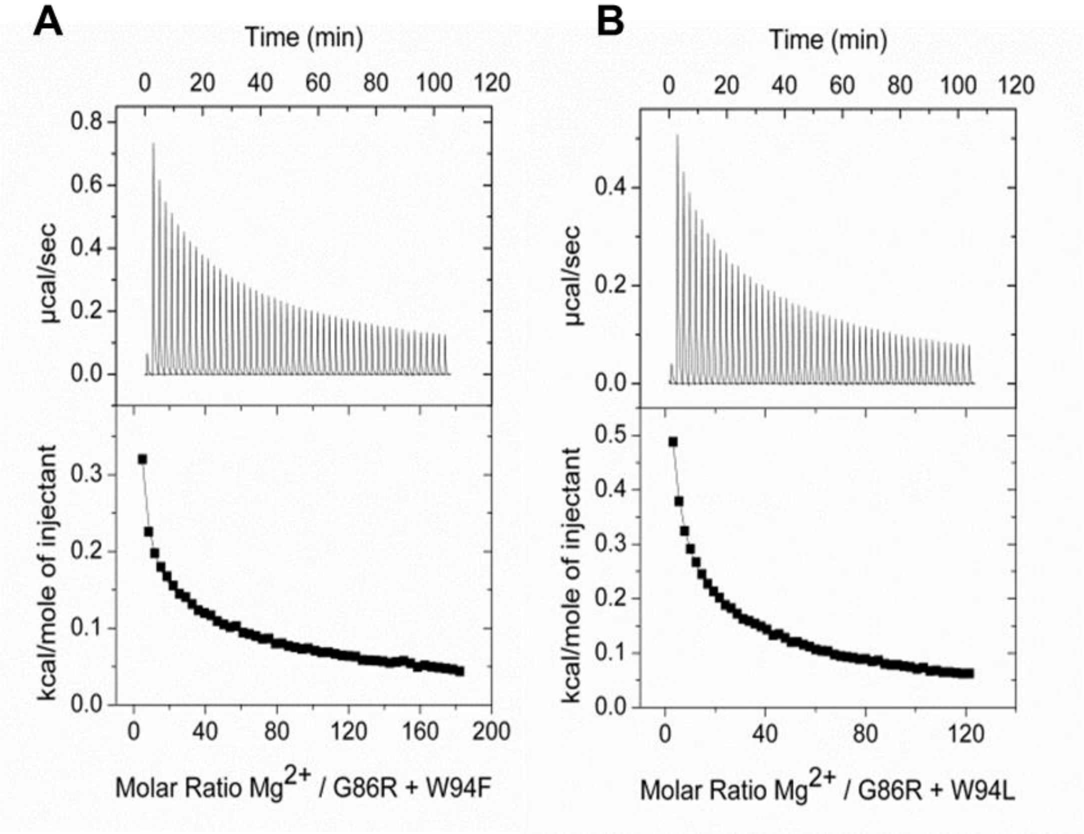

Figure S2. Mg^{2+} binding to GCAP1 variants. Representative of Mg^{2+} titrations of 20 μM GCAP1 + W94F shown in panel A and GCAP1 + W94L shown in panel B. The Mg^{2+} titration data was fitted with 2-site-binding model yielding K_D1 and K_D2. The dissociation constants (K_D) and enthalpy changes (ΔH) are reported in **Table 1**.

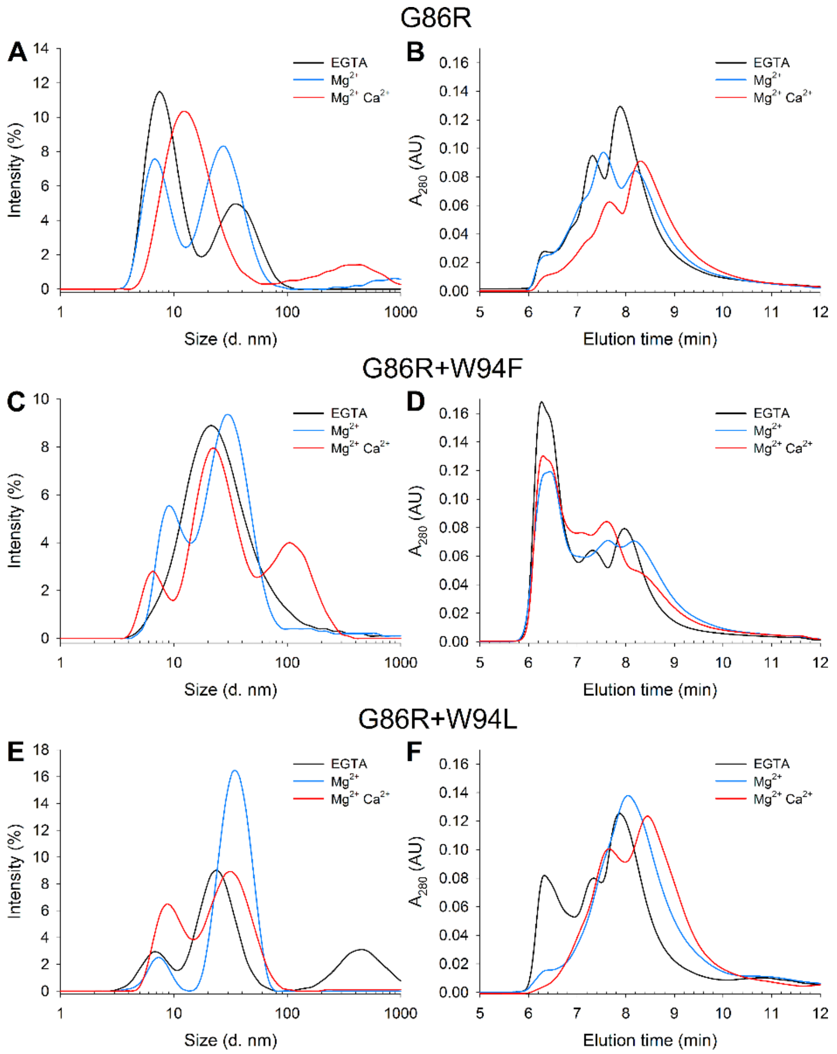

Figure S3. Hydrodynamic diameter estimation of GCAP1 mutants G86R, G86R+W94F and G86R+W94L monitored by Dynamic Light Scattering (A, C, E) and analytical Size Exclusion Chromatography (B, D, F) at different ionic strength. DLS measurements were performed at 37 °C in 20 mM Tris-HCl, 150 mM KCl, 1 mM DTT in the presence of 500 µM EGTA (black), 500 µM EGTA and 1 mM Mg^{2+} (blue) or 500 µM EGTA, 1 mM Mg^{2+} and 1 mM Ca^{2+}(red). Curves represent an average of ~50 measurements, each consisting of 12-15 runs. Analytical SEC measurements were performed at room temperature in 30 mM MOPS pH 7.2, 50 mM KCl, 4 mM NaCl, and 1 mM DTT in the presence of 2 mM EGTA (black), 2 mM EGTA and 3.5 mM Mg^{2+} (blue) or 2 mM Ca^{2+} (red).

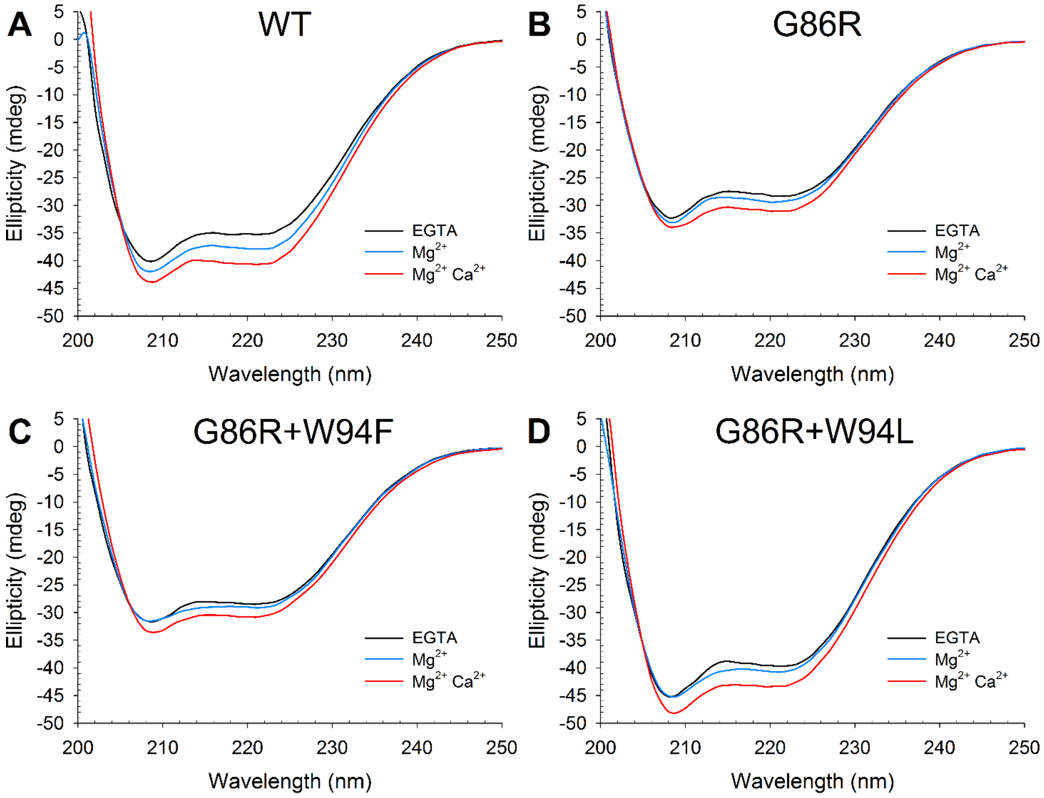

Figure S4. Secondary structure changes occurring in GCAP1 variants upon ion binding, monitored by CD spectroscopy. Far UV CD spectra of ~12 μM GCAP1 WT (A), G86R (B), G86R+W94F (C), G86R+W94L (D) in the presence of 300 μM EGTA (black), 300 μM EGTA and 1 mM Mg^{2+} (blue) or 300 μM EGTA, 1 mM Mg^{2+} and 600 μM Ca^{2+}(red). All experiments were performed at 37 °C in 20 mM Tris-HCl, 150 mM KCl, 1 mM DTT buffer.

Table S1. Geometric descriptors for cation-π interaction monitored by MD simulations. Distances were calculated considering Cα of residues 86, 168 and 178 and Cδ² of residues 21 and 94.

Distance	WT	G86R	WT	G86R
	Ca^{2+}-loaded		EF2/EF3-Mg^{2+}	
**G/R86 - W21**	1.39 ± 0.08	1.27 ± 0.09	1.50 ± 0.15	1.48 ± 0.19
**W21 - W94**	1.19 ± 0.15	0.80 ± 0.05	1.10 ± 0.20	1.01 ± 0.10
**G/R86 - W94**	1.60 ± 0.07	1.23 ± 0.13	1.55 ± 0.08	1.48 ± 0.07
**D168 - R178**	1.09 ± 0.12	1.48 ± 0.21	1.48 ± 0.14	1.40 ± 0.18

© 2020 by the authors. Submitted for possible open access publication under the terms and conditions of the Creative Commons Attribution (CC BY) license (http://creativecommons.org/licenses/by/4.0/).
